# Comparing the effects of reduced social contact on psychosocial wellbeing before and during the COVID-19 pandemic: a longitudinal survey from two Norwegian counties

**DOI:** 10.1007/s11136-023-03350-z

**Published:** 2023-02-11

**Authors:** Jorid Kalseth, Marian Ådnanes, Solveig Osborg Ose, Eva Lassemo, Silje L. Kaspersen, Roshan das Nair

**Affiliations:** 1 Department of Health Research, SINTEF Digital, Trondheim, Norway; 2 School of Medicine, University of Nottingham, Nottingham, UK

**Keywords:** Social contact, Psychosocial wellbeing, Life satisfaction, Longitudinal study

## Abstract

**Purpose:**

To determine changes to people’s social contact during COVID-19, and whether reduced social contact was associated with changes to psychosocial wellbeing.

**Methods:**

Questionnaire data were collected from a sample of adult respondents (18 years or more) in two Norwegian counties participating pre-COVID-19 (September 2019–February 2020; *n* = 20,196) and at two time points during COVID-19 (June [Mid] and November/December [Late] 2020; *n* = 11,953 and *n* = 10,968, respectively). The main outcome measures were participants' self-reported *changes* to social contact, loneliness, psychological distress, and life satisfaction.

**Results:**

The proportion of respondents reporting less social contact due to COVID-19 decreased from 62% in Mid-2020 to 55% in Late-2020. Overall, reported psychological wellbeing remained unchanged or improved from pre-COVID-19 to Mid-2020. From Mid-2020 to Late-2020, however, a reduction in psychological wellbeing was observed. Poorer psychological wellbeing was found for those with less social contact during the pandemic compared with people reporting unchanged social contact. This effect increased over time and was observed for all age groups at Late-2020. At Mid-2020, the importance of change in social contact for change in psychological wellbeing was greatest among young adults (< 30 years), while no significant differences were found for the oldest age group.

**Conclusion:**

The association between COVID-19-era changes to social contact and loneliness, psychological distress, and life satisfaction is complex and appears to be age-dependent. Future studies should consider the quality of social contact and cultural contexts in which social restrictions are imposed.

**Supplementary Information:**

The online version contains supplementary material available at 10.1007/s11136-023-03350-z.

## Introduction

The COVID-19 pandemic and subsequent governmental response in relation to curtailing citizens’ ability to meet others in person has had positive impacts in terms of reducing the spread of the virus and reducing the strain on healthcare systems [1], but has also had a negative impact in terms of people’s health, psychological wellbeing, and quality of life [2–7].

In the COVID-19 era, governments across the world have needed to tread a fine balance between protecting people from a deadly virus, but also enabling them the freedoms to socialise and protect themselves physically, psychologically, and financially. Debates have therefore ensued about finding this ‘sweet spot’ of how much and how fast to ‘open- up’ society [8], and different countries have taken different approaches to this. While most countries opted for ‘lockdowns’ of various intensities, Sweden, for instance, had very few restrictions during the early months of the pandemic [9].

In our Norwegian context, the first COVID-19 case was reported on 21st February 2020 [10]. The government enforced a national ‘lockdown’ on 12th March 2020 and introduced the strictest measures in Norway since World War II [11, 12]. Social contact outside households during this time was significantly curtailed, and there was considerable social discourse in newspapers and the television about the possible psychological consequences of the cut down of such social contact. In a review of change in social contact patterns during the initial mitigation period in the spring of 2020, including eleven studies in European countries, most studies reported between 2 and 5 contacts outside of home per person per day, while pre-COVID rates ranged from 7 to 26 contacts per day [13].

Pre-COVID research studies have shown that social isolation and loneliness negatively affect people both mentally and physically [14]. In fact, a meta-analytic study [15] found that loneliness and social isolation were risk factors for early mortality, with social isolation, loneliness, and living alone corresponding to an average of 29%, 26%, and 32% increased likelihood of mortality, respectively. Conversely, studies have shown that those experiencing more in-person contact were less likely to have poorer psychological wellbeing [16]. Furthermore, studies show that social support, which is predicated on social contact, is a strong predictor of resilience following large-scale disasters, such as earthquakes [17].

Our overall objective, therefore, was to determine whether there were any changes to people’s social contact, and whether reduced social contact from before the pandemic to two time points during the pandemic was associated with changes to psychological wellbeing (in terms of loneliness, psychological distress, and life satisfaction) in two Norwegian counties. A second objective was to determine whether there were age-related differences in these changes.

## Materials and methods

### Survey and participants

Our data were drawn from a sample of adult respondents (18 years or more) from two Norwegian counties participating in a pre-COVID-19 Norwegian Counties Public Health Survey (NCPHS) in Late-2019/Early-2020, including 28,047 respondents from the county of Agder (46% response rate) and 24,222 from Nordland (47% response rate). A random sample of 20,196 respondents were drawn from the pre-Covid NCPHS surveys. These participants were then invited to complete two further rounds of the survey during the pandemic. Therefore, we had three waves of the survey:

- Pre-COVID-19/Late-2019/Early-2020 (Agder: 23 September–18 October 2019, and Nordland: 27 January–16 February 2020), *n* = 20,196.
- Mid-2020 (4–18 June 2020).
- Late-2020 (18 November–4 December 2020).

### Procedure

The NCPHS is a cross-sectional study of health and quality of life in the Norwegian general population, administered by the Norwegian Institute of Public Health, regulated in "Regulations on overview of public health" [18].

The participants are drawn at random from the National Population Register. Email addresses and mobile phone numbers are provided by The Agency for Public Management and eGovernment (Difi). Links to the survey were distributed by email and SMS.

Participation in the survey is based on the consent of the participants. SINTEF’s participation and arrangement for storing the data were approved by the Norwegian Center for Research Data (no. 784440).

### Measures

The study included three different measures to capture different aspects of psychological wellbeing at pre-COVID-19, Mid-2020, and Late-2020, and one measure of changes to social contact due to COVID-19 measured at Mid-2020 and Late-2020.

#### Life satisfaction

To measure participants’ life satisfaction, participants rated the item ‘Overall, how satisfied are you with life these days?’ This is an often-used measure to evaluate subjective wellbeing, for instance, in the European Union’s (EU’s) statistics on income and living conditions (EU-SILC) survey, and is among the core measures of subjective wellbeing recommended by the Organisation for Economic Cooperation and Development [19]. Participants responded on scale ranging from 0 ‘Not at all’ to 10 ‘Very’. However, to be comparable with the other two outcome measures in terms of direction of scale, i.e. higher values signal poorer outcome, we have reversed the scoring of the scale.

#### Loneliness

To measure level of loneliness, participants rated the item ‘Think about the past 7 days. To what degree did you feel lonely?’ on a scale ranging from 0 ‘Not at all’ to 10 ‘Very’. This measure is included in the minimum list for measuring affect in the recommended measurement system for quality of life [20].

#### Psychological distress

The five-item Hopkins Symptom Checklist (HSCL-5) [21] was used to measure participants’ psychological distress. This has been used with other Norwegian samples and psychometric properties reported [22]. The items capture feeling of fearfulness, nervousness, hopelessness, feeling blue, and feeling worried (in the last week). The items were rated on a scale from 1 ‘Not at all’ to 4 ‘Very’. To apply the same statistical approach on all three outcome measures, the sum of scores on the five items, rather than a cut-off point, was used in this study, i.e. range from 5 to 20, with higher scores indicating more distress. Furthermore, to have all three outcome measures on a common scale, the sum score was rescaled to values in the range from 0 to 10 using the min–max algorithm (cf. supplementary material).

#### Change in social contact

Change in social contact due to COVID-19 was measured by the item ‘How has the corona situation affected your social contact with others (including telephone contact and digital contact)?’ This item was measured at Mid-2020 and Late-2020 and had three mutually exclusive response alternatives: ‘More contact with others’, ‘Unchanged contact with others’, and ‘Less contact with others’. Only 3.6% of the participants answered ‘More contact with others’. To keep with our focus on ‘reduced’ social contact, we merged these observations with the unchanged contact group and concentrated on comparison between those with less social contact (given the value 1) to those with more or unchanged social contact (value = 0).

### Statistical analysis

Since the social contact measure was formulated as *change* in social contact due to COVID-19 and was only measured at Mid-2020 and Late-2020, we analysed *change* in psychological wellbeing from before COVID-19 to Mid-2020 and Late-2020, respectively. Therefore, we have an unbalanced panel dataset with observations at two time points, Mid-2020 and Late-2020. The panel is ‘unbalanced’ since not all individuals that were invited participated in both Mid-2020 and Late-2020 surveys. Linear mixed (multilevel) multivariate change score regressions with random intercept for individuals were used to analyse the association between change in social contact due to COVID-19 and change in psychological wellbeing, considering that individuals (level 2) can participate at two times (level 1). The mixed model approach combines the between-subject and within-subject variation in the data [23]. Change score analysis allows us to study change in psychological wellbeing in relation to reported chance in social contact due to COVID-19. The outcome variables are measured as the change in wellbeing scores (life satisfaction, loneliness, and psychological distress) from before COVID-19, i.e. as the difference between the score at Mid-2020 and Late-2020, respectively, and the score pre-COVID-19. As pointed out by Hansen et al. [24], who used the NCPHS-data to study change in loneliness from before COVID-19 to Mid-2020, change score analysis is appropriate when there is a strong negative correlation between initial status and change in the outcome measure [25], which is the case in our data.

The response to the COVID-19 situation for perceived change in social contact and for psychological wellbeing may have changed during different phases of COVID-19. To allow the effect of less social contact on psychological wellbeing to vary from Mid-2020 to Late-2020, interactions between social contact and measurement occasion (time) were included in the regressions. The model specification is included as supplementary material.

Because the relationship between change in social contact and change in psychological wellbeing may differ between population groups [26], we investigated the differences between age groups; therefore, all analyses were stratified by age. We grouped the participants into four age groups: < 30, 30–49, 50–64, 65 + and performed separate analyses for each group. See supplement for more details on model and variable specifications. Furthermore, since the relationship between change in social contact and change in psychological wellbeing may be affected (mediated) by other respondent characteristics, analyses without (unadjusted results) and with control (adjusted results) for socio-demographic characteristics (covariates) were performed and results compared.

#### Covariates

We controlled for socio-demographics likely to be associated both with the outcome measures and change in social contact due to COVID-19 [24], i.e. sex, education, relationship status, and employment status. Furthermore, we also controlled for level of participation in organised leisure activities pre-COVID-19 and working from home during Mid-2020 and Late-2020, since these variables were likely to affect the probability to change behaviour related to social contact due to lockdown restrictions (please see Table 1 for variables). Age, sex, education, and leisure activity participation were measured at pre-COVID and are therefore constant, while the other variables were measured at both Mid-2020 and Late-2020.

**Table 1:** Descriptive statistics, and changes in life satisfaction, loneliness, and life satisfaction for Mid-2020-survey and Late-2020-survey, total and by age groups

	Mid-2020					Late-2020				
	Age groups					Age groups				
	< 30	30–49	50–64	65 +	Total	< 30	30–49	50–64	65 +	Total
N	1088	3763	4386	2716	11,953	899	3275	4151	2643	10,968
%	9.1	31.5	36.7	22.7	100.0	8.2	29.9	37.8	24.1	100.0

	< 30	30–49	50–64	65 +	Total	< 30	30–49	50–64	65 +	Total
	%	%	%	%	%	%	%	%	%	%
**Social contact**										
More	6.6	4.5	3.3	3.8	4.1	6.0	3.3	2.8	2.5	3.1
Unchanged	41.7	31.8	32.3	34.6	33.5	38.6	37.5	40.8	49.4	41.7
Less	51.7	63.7	64.4	61.6	62.4	55.4	59.3	56.4	48.1	55.2
Total	100.0	100.0	100.0	100.0	100.0	100.0	100.0	100.0	100.0	100.0
**Sex**										
Female	65.8	59.4	54.5	43.0	54.5	64.5	59.0	53.0	42.5	53.2
Male	34.2	40.6	45.5	57.0	45.5	35.5	41.0	47.0	57.5	46.8
Total	100.0	100.0	100.0	100.0	100.0	100.0	100.0	100.0	100.0	100.0
**Partner**										
Married/cohabiting	44.6	79.5	77.5	75.7	74.7	48.3	78.8	77.5	75.5	75.0
Non-cohabiting partner	15.1	4.9	5.2	4.5	5.8	11.1	4.3	5.4	4.9	5.4
Single	40.3	15.6	17.3	19.8	19.4	40.5	16.9	17.1	19.6	19.5
Total	100.0	100.0	100.0	100.0	100.0	100.0	100.0	100.0	100.0	100.0
**Education**										
Low	8.1	6.8	12.4	19.3	11.8	7.9	6.2	12.7	19.4	12.0
Medium	48.9	31.4	37.8	32.7	35.6	47.6	31.2	37.7	32.4	35.3
High	43.0	61.8	49.8	48.1	52.6	44.5	62.6	49.6	48.2	52.7
Total	100.0	100.0	100.0	100.0	100.0	100.0	100.0	100.0	100.0	100.0
**Employment**										
Employed	52.7	80.7	72.1	9.4	58.9	55.9	82.4	69.6	8.5	57.6
Temporary laid off	4.0	3.2	1.9	0.3	2.1	1.3	1.0	1.0	0.1	0.8
Unemployed	3.7	1.4	0.9	0.1	1.2	3.0	1.6	1.0	0.1	1.1
Pupil/Student	32.1	2.3	0.2	0.0	3.7	32.0	2.1	0.2	0.0	3.3
Other	7.5	12.4	24.9	90.2	34.1	7.8	12.8	28.2	91.3	37.1
Total	100.0	100.0	100.0	100.0	100.0	100.0	100.0	100.0	100.0	100.0
**Home office**										
No	85.4	75.7	77.2	96.7	81.9	94.1	90.5	89.9	98.6	92.5
Yes	14.6	24.3	22.8	3.3	18.1	5.9	9.5	10.1	1.4	7.5
Total	100.0	100.0	100.0	100.0	100.0	100.0	100.0	100.0	100.0	100.0
**Leisure activities**										
Weekly	23.1	37.4	29.2	33.5	32.2	24.0	37.0	29.1	33.8	32.2
1–3 times a month	10.8	16.1	15.1	17.7	15.6	11.3	15.7	15.3	17.5	15.6
Less often	29.5	27.4	33.0	29.3	30.1	29.1	28.3	33.2	29.7	30.5
Never	36.6	19.1	22.7	19.6	22.1	35.5	19.1	22.4	19.0	21.7
Total	100.0	100.0	100.0	100.0	100.0	100.0	100.0	100.0	100.0	100.0
Life satisfaction* (change from pre-COVID-19, scale reversed)										
Mean^a^	0.03	− 0.09	− 0.04	0.11	− 0.02	0.59	0.36	0.50	0.62	0.49
Standard deviation	1.94	1.67	1.62	1.57	1.65	2.09	1.85	1.80	1.69	1.82
**Loneliness (change from pre-COVID-19)**										
Mean^a^	− 0.12	− 0.31	− 0.19	0.06	− 0.17	0.67	0.27	0.46	0.70	0.48
Standard deviation	2.84	2.46	2.36	2.21	2.42	3.08	2.64	2.70	2.50	2.68
**Psychological distress (change from pre-COVID-19)**										
Mean^a^	− 0.47	− 0.36	− 0.33	− 0.17	− 0.32	0.27	0.17	0.11	0.23	0.17
Standard deviation	2.27	1.75	1.45	1.25	1.60	2.22	1.84	1.54	1.33	1.66

Number of respondents (*N*) with non-missing values varies from 11,333 to 11,953 at Mid-2020 and from 10,502 to 10,968 at Late-2020

^a^Mean change score for all respondents over all age groups are significant different from zero (*p*-value < 0.01) for all measures and both time points, except for change in life satisfaction at Mid-2020. The latter result applies to age groups < 30 years and 50–64 years. Also change score for loneliness at Mid-2020 are not significantly different from zero for age groups < 30 and 65+years

*Life satisfaction variable is reverse scored

Analyses were conducted using STATA version SE 16.1. The *mixed* command was used to perform linear mixed (multilevel) multivariate analyses. We have used the STOBE guidelines [27] to report our study.

## Results

**Fig. 1:**
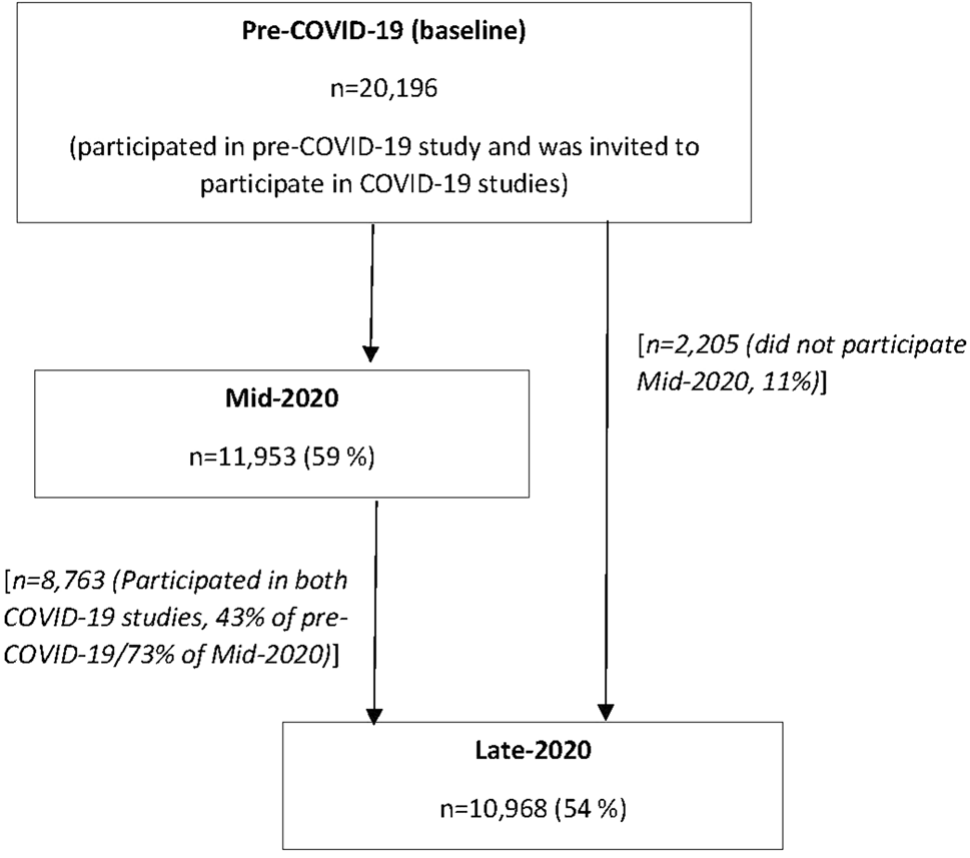
The number of respondents for each wave of the survey and response rates

In total, 11,953 (59% response rate) and 10,968 (54% response rate) people responded for Mid-2020 and Late-2020 of NCPHS COVID-19 study, respectively. 8,763 (43% response rate) participated in all three waves of the survey (see Fig. 1).

Approximately three-quarters of the respondents were married or cohabiting; however, this was less than half among respondents under 30 years. More than half had tertiary education, varying between age groups from about 40% among the youngest and about 60% in the age group 30–49 years. Nearly 60% were employed, varying from about 80% in the age group 30–49 years to less than 10% for those aged 65 or older. Approximately a third reported participating in organised activities on a weekly basis and just above a fifth reported never participating in organised activities pre-COVID-19. Younger people participated in organised leisure activities less often than the older age groups. Nearly one in four in ages 30 to 64 worked from home in Mid-2020, and this fell to about one in ten in Late-2020 (Table 1). For pre-COVID descriptive statistics, see Hansen et al. [24].

Most of the respondents reported having less social contact during the pandemic than before (Table 1); however, the numbers reporting less social contact went down from 62% in Mid-2020 to 55% in Late-2020. This corresponds with the share of respondents reporting working from home, which declined from 18% in Mid-2020 to 7% in Late-2020. The largest decrease in the share reporting less social contact from Mid-2020 to Late-2020 were found for the oldest group, 65 years or older, with a reduction from 62 to 48%. Hence, at Late-2020, this group had the lowest share of respondents reporting less social contact. At Mid-2020, the percentage reporting less social contact was lowest among young people under 30 years, 52%, i.e. 10% points or more below the share for the older age groups. The percentage reporting less social contact *increased* from Mid-2020 to Late-2020 among the youngest age group, from 52 to 55%.

While there were no changes in overall mean score for life satisfaction from pre-COVID-19 to Mid-2020, all other change scores were significantly different from zero (Table 1). Hence, there was no overall change in life satisfaction and a reduction in reported loneliness and psychological distress from pre-COVID-19 to Mid-2020. However, there was a small but significant increase in life satisfaction for the 30–49 age group, and a small significant negative change in life satisfaction for the 65 + age group. For loneliness, no significant change of mean scores was observed for the age groups < 30 and 65 + years at Mid-2020.

**Fig. 2:**
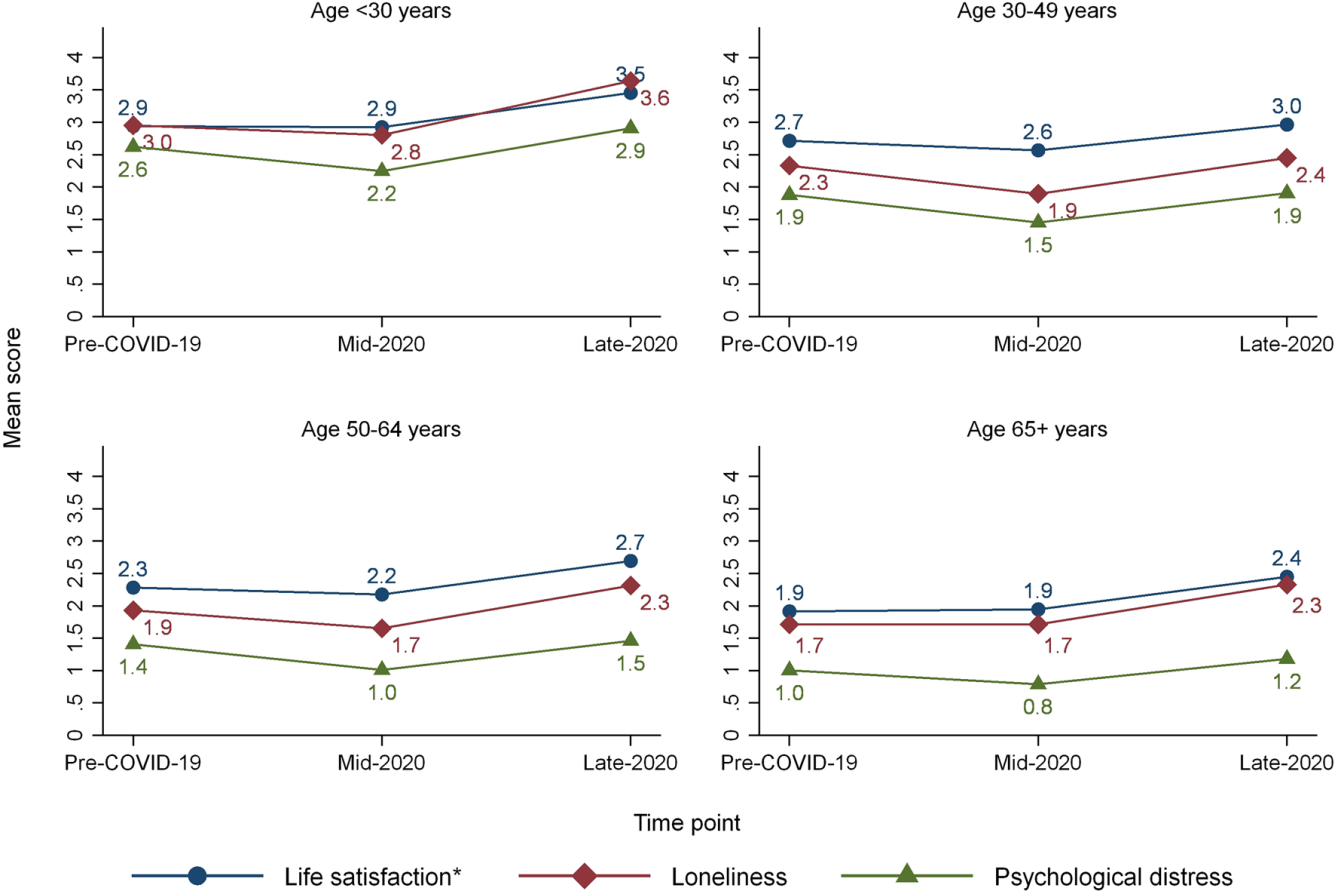
Mean score for life satisfaction (*scale reversed), loneliness and psychological distress pre-COVID-19 (for those participating in either Mid-2020 or Late-2020), Mid-2020 and Late-2020, by age group. *The original scale of life satisfaction is reversed in the analyses

The overall change from pre-COVID-19 to Late-2020, however, demonstrated a reduction in life satisfaction and an increase in loneliness and psychological distress. This result was found for all age groups (see Fig. 2). Compared to the variation in reported changes among respondents, the mean change in outcome scores was modest (0.2 to 0.5 on a 0 to 10 scale) (Table 1). [Fig. 2].

Despite fewer people reporting a reduction in social contact (except for the youngest age group), we found poorer outcomes related to psychological wellbeing at Late-2020 than Mid-2020. This therefore requires a separate explanation. However, we find a pattern of poorer psychological wellbeing outcomes for those reporting less social contact due to COVID-19 than those reporting more or unchanged social contact (Table 2). For example, at Late-2020, 52% of respondents reporting less social contact had poorer life satisfaction scores, compared to 40% among those reporting more or unchanged social contact. Similarly, at Late-2020, the share of respondents with increased loneliness and psychological distress was 9 and 12 percentage points higher, respectively, for those reporting less social contact compared to more or unchanged social contact.

**Table 2:** Distribution of respondents by change in psychological wellbeing (life satisfaction, loneliness, and psychological distress) from pre-COVID-19 at Mid-2020 and Late-2020

	Mid-2020				Late-2020			
	Total % (*n*)	Unchanged or more social contact % (*n*)	Less social contact % (*n*)	Difference^a^	Total % (*n*)	Unchanged or more social contact % (*n*)	Less social contact% (*n*	Difference^a^
Life satisfaction								
More satisfied with life	32.8 (3871)	35.1	31.4	− 3.7	23.9 (2588)	27.8	20.7	− 7.1
Unchanged life satisfaction	35.4 (4180)	35.6	35.3	− 0.3	29.6 (3207)	32.3	27.4	− 4.9
Less satisfied with life	31.8 (3751)	29.3	33.3	4.0	46.5 (5041)	39.9	51.9	12.0
Total	100.0 (11,802)	100.0 (4436)	100.0 (7366)		100.0 (10,836)	100.0 (4850)	100.0 (5986)	
Loneliness								
Less lonely	32.8 (3708)	35.2	31.3	− 3.9	25.6 (2677)	27.8	23.8	− 4.0
Unchanged loneliness	42.1 (4764)	41.8	42.3	0.5	35.8 (3749)	38.6	33.6	− 5.0
More lonely	25.1 (2842)	23.0	26.4	3.4	38.6 (4044)	33.6	42.7	9.1
Total	100.0 (11,314)	100.0 (4233)	100.0 (7081)		100.0 (10,470)	100.0 (4676)	100.0 (5794)	
Psychological distress								
Less distressed	39.2 (4581)	41.6	37.8	− 3.8	28.8 (3079)	31.0	27.0	− 4.0
Unchanged distress	39.2 (4583)	39.8	38.8	− 1.0	34.7 (3704)	39.2	31.0	− 8.2
More distressed	21.6 (2524)	18.6	23.4	4.8	36.5 (3899)	29.8	42.0	12.2
Total	100.0 (11,688)	100.0 (4415)	100.0 (7273)		100.0 (10,682)	100.0 (4792)	100.0 (5890)	

Percentages. Total and by change in social contact due to COVID-19. *n* = number of respondents. Pearson's χ2 tests indicate significant differences in distribution of respondents between groups for all three psychological wellbeing measures at both Mid-2020 and Late-2020

^a^Difference between social contact groups, i.e. the two preceding columns = per cent of respondents with higher/unchanged/lower wellbeing score among those with less social contact due to COVID-19 *minus* per cent of respondents with higher/unchanged/lower wellbeing score among those with unchanged/more social contact due to COVID-19

A similar pattern was observed at Mid-2020, however, with smaller differences between the groups. For example, in Mid-2020, 33.3% reported ‘less satisfied with life’ if they reported reduced social contact, while only 29.3% of people reported so if social contact was unchanged or increased. Further, while 35.2% and 41.6% reported to be less lonely and less distressed (respectively) at Mid-2020 among those with more or unchanged social contact, the corresponding percentages were only 31.3% and 37.8% among those with less social contact.

**Fig. 3:**
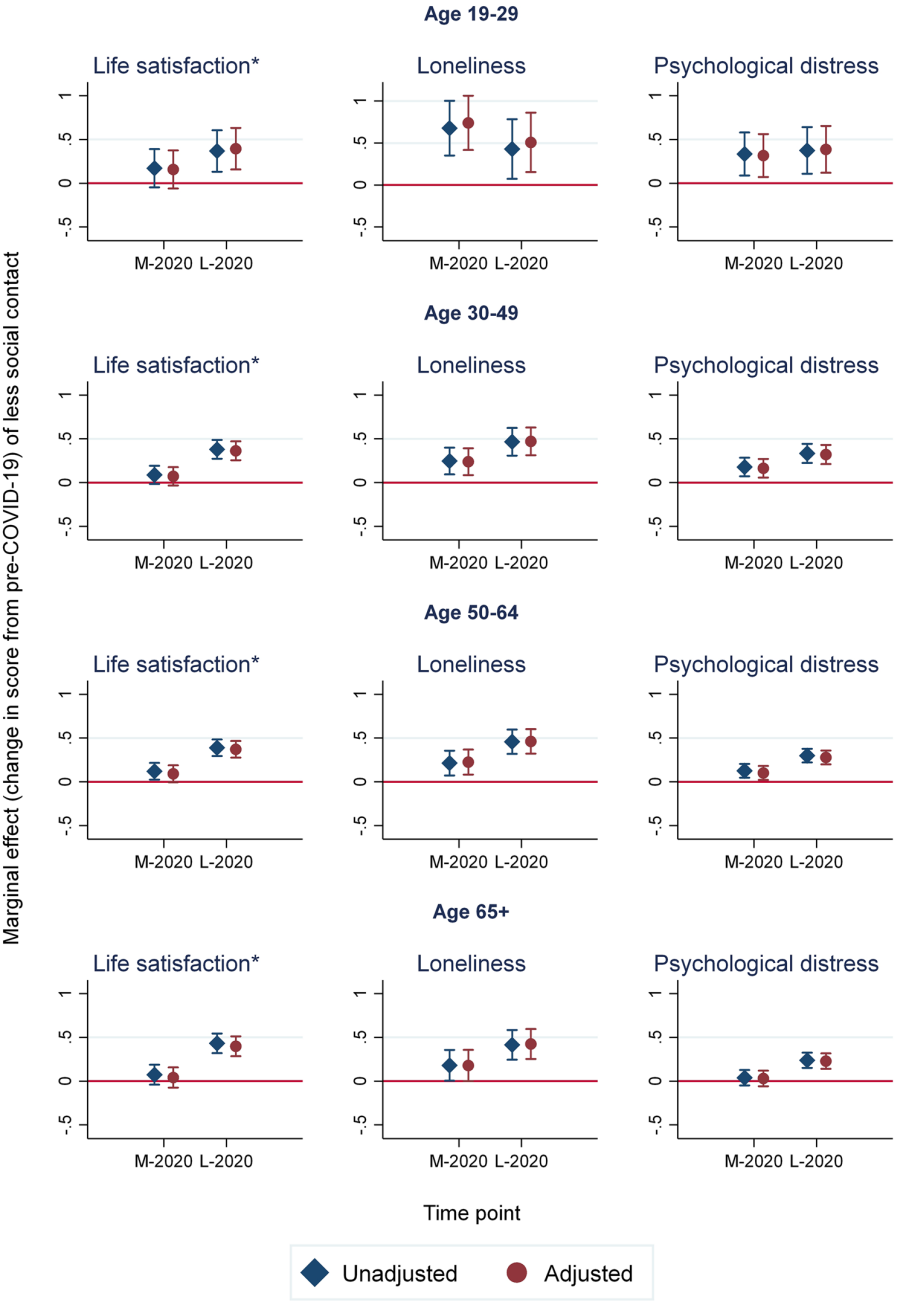
Age group-specific results for *less* social contact (*coefficient and 95% confidence interval)* of the linear mixed (multilevel) *change score* regression analyses, unadjusted and adjusted for covariates. M-2020 and L-2020 indicate results for effect of social contact variable on change from pre-Covid-19 to, respectively, Mid-2020 (M) and Late-2020 (L). Confidence interval crossing the red line indicates zero effect

Figure 3 shows the result for the variable "less social contact" from the change score regression analysis for each of the four age groups. Full results from the regression analyses by age group are included as supplementary material.

Overall, visual inspection of the regression results of Fig. 3 demonstrates similar findings across age groups. However, we found larger effect sizes for less social contact on loneliness [0.68 (SE: 0.17)] and psychological distress (0.33 [SE: 0.13]) for the youngest group (age 19–29) at Mid-2020. Furthermore, there were no significant differences on loneliness (0.18 [SE: 0.09]) and psychological distress (0.039 [SE: 0.045]) for less social contact compared to unchanged or more social contact for the oldest group (age 65 +) in the early phase of COVID-19. The inclusion of covariates did not affect the results for the effect of change in social contact.

### Change in life satisfaction

There was no difference in the change in life satisfaction from pre-COVID-19 to Mid-2020 between those reporting less social contact and more or unchanged social contact. This, however, changed at Late-2020. For all age groups, individuals reporting less social contact had a larger decrease in life satisfaction at Late-2020 compared to pre-COVID-19 than individuals reporting more or unchanged social contact. The effect sizes of changed social contact on life satisfaction were modest compared to variation in overall change in the outcome among respondents, with standardised effect sizes at Late-2020 ranging between 0.18 and 0.26.

### Change in loneliness

Overall, less social contact was associated with less decrease in loneliness from pre-COVID-19 to Mid-2020 and greater increase in loneliness at Late-2020, when compared to those reporting more or unchanged social contact. However, for those under 30 years of age, the effect of reduction in social contact on psychological wellbeing was larger at Late-2020 than in Mid-2020. However, measured relative to standard deviation of change in scores by age group, the effect size of less social contact on loneliness was modest, less than 0.10 at Mid-2020 [except for the youngest age group (0.24)], and in the range from 0.14 to 0.18 at Late-2020.

### Change in psychological distress

Overall, those reporting more or unchanged social contact had better outcomes in terms of psychological distress than those reporting less social contact. Less social contact was associated with less decrease in psychological distress from pre-COVID-19 to Mid-2020 and a higher increase in psychological distress at Late-2020. Except for those under 30 years, for whom the effect was the same at both Mid-2020 and Late-2020, we found that less social contact had a larger impact on psychological distress at Late-2020 than at Mid-2020 for all other age groups. At Mid-2020, the estimated effect was close to zero and non-significant for the 65 + age group. Measured relative to overall standard deviation of change at Mid-2020 for each age groups, the effects size was about 0.10 for the age groups from 30 to 64 years, and 0.15 for the ages under 30 years. At Late-2020, the standardised effect sizes were between 0.17 and 0.19.

For those reporting less, more or unchanged social contact, outcomes at Mid-2020 were unchanged or improved compared to pre-COVID-19 for all three measures. For those reporting less social contact, outcomes at Late-2020 were poorer compared to pre-COVID-19 for all three measures. For those reporting more or unchanged social contact at Late-2020, they were either back to the same level as before COVID-19 or had poorer outcomes.

## Discussion

In this study, we wanted to determine whether there were any changes to people’s social contact, and whether reduced social contact from before the pandemic to two time points during the pandemic was associated with changes to psychological wellbeing (in terms of loneliness, psychological distress, and life satisfaction) in two Norwegian counties, and to determine whether there were age-related differences in these changes. Our findings show that overall, across all age groups (> 18 years), with some differences, people had less social contact with others during the pandemic than before. Overall, reporting less social contact due to COVID-19 was associated with poorer psychological wellbeing compared to reporting more or unchanged social contact.

### Social connectedness during the pandemic

Social connectedness is crucial for mental health and wellbeing [28]. Indeed, like in our study, another Norwegian study [29] (*n* = 8676) at the start of the pandemic found that between 74 and 84% had reduced or avoided taking public transport, going to shops or to public events, thereby significantly reducing social contact.

### Time trends in effects of the pandemic

We found no overall change in life satisfaction and a reduction in reported loneliness and psychological distress from pre-COVID-19 to Mid-2020. This is in line with findings from a study from England (Fancourt et al. [30]), which found that anxiety and depression levels declined over the first 20 weeks following the introduction of lockdown. The authors suggested that this might be because people “adapted to circumstances” This might be the case with our cohort also. Another reason that we did not find a more negative effect on psychological wellbeing from pre-COVID-19 to Mid-2020 is perhaps because people felt that this was a universal condition affecting everyone and that we were all in it together [31], with the pandemic having created a level playing field, especially for those who had experienced loneliness or had few friends as the pre-pandemic ‘norm’. Indeed, based on the same pre-COVID-19 and Mid-2020 data as our study, Hansen et al. [24] found that overall loneliness was “stable or falling during the lockdown”, i.e. during the early part of the pandemic. Furthermore, one review [32] found that life satisfaction and loneliness remained "largely stable" throughout the initial year of the pandemic. Finally, the Mid-2020-survey was conducted during a phase of easing up of national restrictions which could have also impacted the results.

However, we did find that as the pandemic and its impact continued, the overall change from Mid-2020 to Late-2020 demonstrated a *reduction* in life satisfaction and an increase in loneliness and psychological distress. Such time trends have also been reported by others. For example, a Danish study [33] showed a similar time trend, with the worst levels of loneliness being observed during the second lockdown (i.e. Late-2020/Early-2021). In our study, the Late-2020 survey occurred during tightening of restrictions in Norway. The Danish study also found poorer mental health being observed during the strictest phases of the lockdowns, with this lifting as the society opened up more. Some of their other findings, however, were divergent to ours, and such country-specific variations have been reported by others and has been associated with the timing and stringency of the lockdowns, trust in the government, availability of tests, etc. [34].

### Association between social contact and psychological wellbeing

We found that reduced social contact due to COVID-19 was associated with increased loneliness and psychological distress, and lower life satisfaction compared to unchanged social contact. Therefore, less social contact appeared to dampen the positive change in psychological wellbeing measures during the first months of COVID-19 and magnify the negative change during the latter period covered in our study. On the face of it, this appears intuitive and is consistent with findings from studies of the role of social support for psychological wellbeing during COVID-19 across the world [6, 35, 36]. Even social contact with strangers appears to enhance wellbeing [37].

Our study found modest effect sizes of less social contact on psychological wellbeing [38]. Still, differences in the percentage with a reduction of psychological wellbeing outcomes between respondents with reduced social contact and those with unchanged or more social contact were 9–12% points across age groups in Late-2020. Considering that the impact of the COIVD-19 pandemic on psychological wellbeing appears to be complex and time dependent, small effect sizes for mean change in outcome scores are not surprising.

Another Norwegian survey in May 2020 [39] found that more than a quarter of their respondents reported current psychological distress over the threshold for clinically significant symptoms. We found that less social contact due to COVID-19 was associated with increased psychological distress, with lower decrease (Mid-2020) or larger increase (Late-2020) in psychological distress among those who reported less social contact than among those who reported unchanged social contact. Similar issues have been reported in other studies [40–42]. A study from Lebanon [35] found that the risk for depression was 63% lower compared to those with low “perceived social support” (OR = 0.37 [95% CI 0.21–0.67) when adjusted for age, gender, living arrangement, education level, and the presence of chronic conditions or illnesses.

Blix et al. [39] found that a higher level of COVID-related worry was significantly associated with a higher level of psychological distress, and a lower level of life satisfaction, even when adjusting for vulnerability factors. This correspond to our results for social contact, if reducing social contact is interpreted as a signal of level of COVID-related worries. Change in social contact pattern may be influenced by COVID-related worries [43, 44].

One surprising finding was that even though fewer people reported a reduction in social contact due to COVID-19 from Mid-2020 to Late-2020, except for people under 30 years, they reported poorer outcomes at Late-2020 than Mid-2020. One reason could be that people's expectations were related to the easing of restrictions in the autumn of 2020, and resultant disappointment when ‘normalcy’ was not resumed [45]. This might also be associated with ‘pandemic fatigue’ and emotional exhaustion following several months of the pandemic and the resultant changes to people’s lives.

### Age-dependent effects on psychological wellbeing

As highlighted in the previous sections, we found age-related differential effects on social contact and psychological wellbeing. Previous studies have also found wellbeing during COVID-19 to vary with age. For instance, a 4-country (UK, Norway, USA, Australia) survey [46, 47] among adults found that *lower age* was associated with *higher levels* of emotional distress, poorer quality of life and higher feelings of loneliness. Such findings have also been reported in a review of European studies of COVID-19 mental health [6]. Our study shows that the age difference in *change* in life satisfaction, loneliness, and psychological distress was not uniform and varied over time. In line with the finding of Hansen et al. [24] that *older* women reported “slightly increased loneliness” during lockdown, we found that the oldest (65 +) and the youngest (< 30) age groups showed the poorest results, i.e. largest deterioration in the three wellbeing measures at Late-2020 compared to pre-COVID-19. Furthermore, from pre-COVID-19 to Mid-2020 (i.e. during the spring 2020), the importance of change in social contact for change in psychological wellbeing was greatest among young adults (< 30 years) while no significant difference was found for the oldest age group. Similarly, a systematic review on the impact of the COVID-19 lockdown on European university students' negative emotional symptoms found that “isolation, reduced social contact, duration of quarantine and restrictions”, all characteristics of a lockdown, played an important role in increased negative emotional symptoms for this group [5].

Data from Geirdal et al.'s 4-country study (mentioned above) ([46, 47] found that all the countries’ samples (except the Australian one) reported a significantly higher level of loneliness between April and November 2020. This is in accordance with the finding of increased loneliness in individuals from Mid-2020 to Late-2020 in our study. A Dutch study of older adults [48] also found that participants experienced increased loneliness (but mental health remained stable) during the pandemic compared to before. Also, Hansen et al. [24] found that while overall loneliness was stable or falling during the lockdown, some subgroups reported “slightly increased” loneliness. This diversity in responses is noteworthy in our study also - especially differences according to change in social contact.

### Intra-group variability

One interesting finding from the extant literature, which we have alluded to in our discussion, is the intra-group variability seen within the samples studied in different countries. Ours and other studies have considered age- and gender-related differences [7, 49], while others have identified differences in household composition as affecting people’s mood [3]. Therefore, it is unsurprising that studies have also reported certain ‘protective’ factors and ‘psychosocial resources’ that have helped maintain good quality of life during social distancing imposed by governments during the pandemic. For example, a Finnish study found that absence of loneliness and better stress-coping ability increased the odds for constant high quality of life from before to amid social distancing [50]. Therefore, taking a more nuanced approach to understanding population vulnerabilities and psychosocial resources will enable better public policy (e.g. how social distancing is mandated) and provide avenues for better healthcare resource allocation (e.g. in targeting psychosocial interventions) in the future.

### Strengths and limitations

The major strengths of our study were the large sample size allowing us to explore several characteristics, and a design that enabled assessment of change at person level from before COVID-19 and at two timepoints in 2020 (June and November). Hansen et al. [24], studying the same dataset at pre-COVID-19 and Mid-2020, refer to a previous study concluding that online questionnaires are useful in avoiding social desirability bias and in improving reliability to answering sensitive questions [51]. However, online questionnaires are likely to exclude some groups, for example the oldest and those with severe/serious health problems or living in long-term care facilities. Hansen et al. [24] refer to the same combined response rate for Mid-2020 as in our study, i.e. 27%, and our study showed a response rate at 20% for responding at both Mid-2020 and Late-2020. Therefore, non-respondents and dropouts may have affected the generalisability of the findings.

Another limitation is that we did not collect all types of social contacts at all three time points and therefore could not compare certain changes (e.g. between in-person contacts via telephone or other digital devices). Interestingly, researchers have also found that ‘digital contact’ does not promote wellbeing while face-to-face contact does [52]. However, these studies were not conducted in Norway, and we know that there are country-specific variations in terms of amount of social contact and loneliness that people experience and that these findings are age-dependent [26]. Indeed, while modern communication technologies, particularly social media, have allowed people to remain in contact with others, studies have also found that spending more time daily on social media was associated with higher levels of ‘emotional’ loneliness [53]. Future studies could examine whether (and the degree to which) these types of contacts were able to compensate for the lack of in-person social contact.

We were unable to control for seasonal variations, which are known to affect loneliness [54]; pre-COVID-19 was during the Norwegian autumn and winter and Mid-2020 was in the summer, potentially concealing some of the negative emotional impact of COVID-19. Finally, data were collected from two Norwegian counties. We cannot be certain how generalisable these are to the other counties in Norway (or outside). This is an issue for most studies because of the different ways in which the pandemic affected populations (with diverse national mortality rates), the different social and healthcare resources available for populations, and the degree to which different populations value or need high levels of social contact. For instance, Amit Aharon et al. [55] found that Italian participants had a higher level of anxiety symptoms and lower health-related quality of life compared with their Israeli counterparts when assessed at the same timepoints during the pandemic. Any cross-country comparison should therefore be treated with caution.

## Conclusion

The association between social contact and loneliness, psychological distress, and life satisfaction during COVID-19 is a complex one. We found that most respondents reported having less social contact during the pandemic than before; however, the numbers reporting less social contact went down from 62% in Mid-2020 to 55% in Late-2020. Overall, participants' psychological wellbeing remained unchanged or improved from pre-COVID-19 to Mid-2020. From Mid-2020 to Late-2020, however, a reduction in psychological wellbeing was observed. Poorer outcomes in terms of change in psychological wellbeing were found for those with less social contact during the pandemic compared with people reporting unchanged social contact. This effect increased over time and was observed for all age groups at Late-2020. At Mid-2020, the importance of change in social contact for change in psychological wellbeing was greatest among young adults (< 30 years), while no significant differences were found for the oldest age group.

While several studies (including ours) have considered the ‘amount’ of social contact, the ‘quality’ of such contact has largely been unexplored. In addition to local and national contexts such as the severity of the lockdowns or the COVID-19-related casualties, such nuances may explain why there is some discrepancy in findings from different studies. Furthermore, for those who do not live alone, the nature/quality of pre-existing relationships (e.g. marital or family relationships) may also affect the impact of social contact on psychological wellbeing. The exact mechanisms that underlie or drive these associations *and* the rate at (or trajectory with) which these changes occur for the different age groups need further investigation. Indeed, these trajectories might be of real significance to further understand COVID-19-related psychological implications, but also for when and how to provide support where needed.

## Supplementary Information

Below is the link to the electronic supplementary material.

Supplementary file1 (DOCX 65 KB)

## Data Availability

The data that support the findings of this study are available upon application to National Institute of Public Health in Norway. Restrictions are applied to the availability of these data, which were used under license for this study. Application form for access to data is available at: https://www.fhi.no/en/more/access-to-data/elektronisk-soknadsskjema-for-datatilgang/
