# Supplementary material for: Comparing the effects of reduced social contact on psychosocial wellbeing before and during the COVID-19 pandemic: a longitudinal survey from two Norwegian counties
Source: Qual Life Res. 2023 Feb 11;32(6):1771–84. doi: 10.1007/s11136-023-03350-z (PMC9922041; doi:10.1007/s11136-023-03350-z)
Supplement: Supplementary file 1 — Supplementary file1 (DOCX 65 KB) [file 11136_2023_3350_MOESM1_ESM.docx]

**Supplementary material**

1)

**Min-max algorithm**

Min-max algorithm to rescale psychological distress (SCL5) from 5-20 scale to 0-10 scale:

SCL5, rescaled=((10 - 0)/(20 - 5))*(SCL5 - 20) + 10

2)

**Model specification**

The analyses can be specified as in the equation (1)

1. ${dvar}_{it}=\alpha+\beta{\cdot SC}_{it}+ \gamma\cdot T_{t}++\delta{\cdot SC}_{it}\cdot T_{t}+\theta{\cdot X}_{it}+\vartheta{\cdot Z}_{i}+u_{i}+e_{it}$

Where

${dvar}_{it}$= ${var}_{it}-{var}_{ip}$, i.e. score for respectively life satisfaction, loneliness and psychological distress for individual *i* at time *t* (${var}_{it})$*minus* score pre_COVID-19 (${var}_{ip})$, where t=mid-2020, late-2020 and p=pre-COVID-19.

Hence, ${dvar}_{it}$ is the outcome variable for subject i at measurement occasion t, $T_{t}$ is a time indicator for the measurement occasion, ${SC}_{it}$ is measured changed in social contact for individual i at occasion t, $X_{it}$ are other time varying predictor variables, $Z_{i}$ are other predictor variables that does not change with time, $u_{i}$ is unobserved individual random effect and $e_{it}$ is the individual random error term.

3)

**Covariates**

Sex_i_: male, female (reference)

Partner_it_: single, non-cohabiting partner, married or cohabiting (reference)

Education_i_: tertiary, secondary, primary (reference)

Employment_it_: unemployed, temporarily laid off, employed (reference), pupil/student, other

Working from home office_it_: yes, no (reference)

Participated in in organised leisure activities pre-COVID-19_i_: weekly (reference), 1-3 times a month, less often, never

Table S1 Results from the linear mixed (multilevel) change score regressions for life satisfaction, loneliness, and psychological distress, adjusted and unadjusted for covariates for age group < 30 years^a)^.

|  | Life satisfaction^b)^ | | Loneliness | | Psychological distress | |
| --- | --- | --- | --- | --- | --- | --- |
|  | Unadj. | Adjusted | Unadj | Adjusted | Unadj | Adjusted |
|  |  |  |  |  |  |  |
| Social contact=Less (ref.=More/Unchanged) | 0.17 | 0.16 | 0.68*** | 0.74*** | 0.33** | 0.32* |
|  | (0.11) | (0.11) | (0.17) | (0.16) | (0.13) | (0.13) |
| Time=Late-2020 (ref.=Mid-2020) | 0.40*** | 0.38*** | 0.88*** | 0.93*** | 0.66*** | 0.67*** |
|  | (0.11) | (0.11) | (0.16) | (0.17) | (0.12) | (0.12) |
| Interaction: Less social contact • Late-2020 | 0.20 | 0.24 | -0.25 | -0.23 | 0.041 | 0.070 |
|  | (0.16) | (0.16) | (0.23) | (0.23) | (0.17) | (0.17) |
| Sex=Male (ref.=Female) |  | -0.084 |  | -0.13 |  | 0.028 |
|  |  | (0.11) |  | (0.16) |  | (0.12) |
| Partner=Non-cohab. partner (ref.=Married/Cohab.) |  | -0.058 |  | 0.56** |  | 0.23 |
|  |  | (0.14) |  | (0.21) |  | (0.16) |
| Partner=Single (ref.= Married/Cohab.) |  | 0.11 |  | 0.92*** |  | 0.088 |
|  |  | (0.11) |  | (0.16) |  | (0.12) |
| Education=Medium (ref.=Low) |  | -0.45* |  | -0.12 |  | -0.26 |
|  |  | (0.19) |  | (0.28) |  | (0.22) |
| Education=High (ref.=Low) |  | -0.13 |  | -0.097 |  | 0.086 |
|  |  | (0.20) |  | (0.29) |  | (0.22) |
| Employment=Temp.laid off (ref.=Employed) |  | 0.76** |  | 0.44 |  | 0.58 |
|  |  | (0.26) |  | (0.39) |  | (0.29) |
| Employment=Unemployed (ref.=Employed) |  | 0.61* |  | 0.47 |  | 0.030 |
|  |  | (0.25) |  | (0.37) |  | (0.29) |
| Employment=Pupil/Student (ref.=Employed) |  | 0.20 |  | 0.10 |  | 0.32** |
|  |  | (0.11) |  | (0.16) |  | (0.12) |
| Employment=Other (ref.=Employed) |  | 0.043 |  | -0.22 |  | -0.055 |
|  |  | (0.18) |  | (0.27) |  | (0.21) |
| Home office=Yes (ref.=No) |  | -0.13 |  | 0.37 |  | 0.041 |
|  |  | (0.14) |  | (0.21) |  | (0.16) |
| Leisure activities=1-3 times a month (ref.=Weekly) |  | 0.13 |  | -0.84** |  | 0.020 |
|  |  | (0.18) |  | (0.27) |  | (0.20) |
| Leisure activities=Less often (ref.=Weekly) |  | 0.25 |  | 0.085 |  | 0.025 |
|  |  | (0.14) |  | (0.20) |  | (0.16) |
| Leisure activities=Never (ref.=Weekly) |  | -0.27* |  | -0.25 |  | -0.077 |
|  |  | (0.13) |  | (0.20) |  | (0.15) |
| Constant | -0.042 | 0.14 | -0.47*** | -0.75* | -0.62*** | -0.71** |
|  | (0.083) | (0.23) | (0.12) | (0.34) | (0.093) | (0.26) |
|  |  |  |  |  |  |  |
| Observations | 1,980 | 1,976 | 1,962 | 1,958 | 1,955 | 1,950 |
| Number of groups | 1,433 | 1,429 | 1,422 | 1,418 | 1,415 | 1,410 |
|  |  |  |  |  |  |  |
| Test Less +Less•Late-2020=0 (p-value) | 0.002 | 0.001 | 0.018 | 0.005 | 0.006 | 0.005 |
|  |  |  |  |  |  |  |
| AIC | 8195.892 | 8160.896 | 9655.199 | 9604.623 | 8526.053 | 8501.373 |
| BIC | 8229.437 | 8267.083 | 9688.69 | 9710.637 | 8559.522 | 8607.309 |
| Notes: a) Regression coefficients with standard error in parenthesis below coefficient, *** p<0.001, ** p<0.01, * p<0.05. b) The original scale of life satisfaction is reversed in the analyses. | | | | | | |

Table S2 Results from the linear mixed (multilevel) change score regressions for life satisfaction, loneliness, and psychological distress, adjusted and unadjusted for covariates for age group 30-49 years^a)^.

|  | Life satisfaction^b)^ | | | Loneliness | | | Psychological distress | | |
| --- | --- | --- | --- | --- | --- | --- | --- | --- | --- |
|  | Unadj. | Adjusted | | Unadj | Adjusted | | Unadj | | Adjusted |
|  |  |  | |  |  | |  | |  |
| Social contact=Less (ref.=More/Unchanged) | 0.087 | 0.071 | | 0.25** | 0.24** | | 0.18** | | 0.16** |
|  | (0.053) | (0.053) | | (0.078) | (0.078) | | (0.054) | | (0.054) |
| Time=Late-2020 (ref.=Mid-2020) | 0.26*** | 0.28*** | | 0.41*** | 0.42*** | | 0.42*** | | 0.42*** |
|  | (0.054) | (0.054) | | (0.079) | (0.080) | | (0.054) | | (0.055) |
| Interaction: Less social contact • Late-2020 | 0.29*** | 0.29*** | | 0.22* | 0.23* | | 0.16* | | 0.16* |
|  | (0.071) | (0.071) | | (0.10) | (0.10) | | (0.072) | | (0.072) |
| Sex=Male (ref.=Female) |  | -0.14** | |  | 0.047 | |  | | -0.12* |
|  |  | (0.050) | |  | (0.073) | |  | | (0.051) |
| Partner=Non-cohab. partner (ref.=Married/Cohab.) |  | -0.25* | |  | -0.016 | |  | | -0.27* |
|  |  | (0.11) | |  | (0.16) | |  | | (0.11) |
| Partner=Single (ref.= Married/Cohab.) |  | 0.019 | |  | 0.56*** | |  | | 0.088 |
|  |  | (0.065) | |  | (0.096) | |  | | (0.067) |
| Education=Medium (ref.=Low) |  | -0.050 | |  | 0.077 | |  | | 0.14 |
|  |  | (0.10) | |  | (0.15) | |  | | (0.11) |
| Education=High (ref.=Low) |  | 0.0085 | |  | 0.095 | |  | | 0.18 |
|  |  | (0.10) | |  | (0.15) | |  | | (0.11) |
| Employment=Temp.laid off (ref.=Employed) |  | 0.67*** | |  | 0.33 | |  | | 0.56*** |
|  |  | (0.13) | |  | (0.20) | |  | | (0.14) |
| Employment=Unemployed (ref.=Employed) |  | 0.68*** | |  | 0.079 | |  | | 0.41* |
|  |  | (0.17) | |  | (0.24) | |  | | (0.17) |
| Employment=Pupil/Student (ref.=Employed) |  | -0.12 | |  | 0.43* | |  | | -0.15 |
|  |  | (0.14) | |  | (0.20) | |  | | (0.14) |
| Employment=Other (ref.=Employed) |  | -0.47*** | |  | -0.27* | |  | | -0.29*** |
|  |  | (0.072) | |  | (0.11) | |  | | (0.074) |
| Home office=Yes (ref.=No) |  | 0.023 | |  | 0.069 | |  | | 0.0090 |
|  |  | (0.054) | |  | (0.080) | |  | | (0.055) |
| Leisure activities=1-3 times a month (ref.=Weekly) |  | -0.00046 | |  | -0.19 | |  | | 0.12 |
|  |  | (0.072) | |  | (0.11) | |  | | (0.074) |
| Leisure activities=Less often (ref.=Weekly) |  | -0.084 | |  | 0.020 | |  | | -0.037 |
|  |  | (0.061) | |  | (0.089) | |  | | (0.063) |
| Leisure activities=Never (ref.=Weekly) |  | -0.24*** | |  | -0.28** | |  | | -0.065 |
|  |  | (0.069) | |  | (0.10) | |  | | (0.072) |
| Constant | -0.15*** | 0.030 | | -0.47*** | -0.58*** | | -0.47*** | | -0.55*** |
|  | (0.044) | (0.11) | | (0.064) | (0.17) | | (0.045) | | (0.12) |
|  |  |  | |  |  | |  | |  |
| Observations | 7,008 | 6,979 | | 6,884 | 6,858 | | 6,924 | | 6,895 |
| Number of groups | 4,489 | 4,472 | | 4,434 | 4,419 | | 4,441 | | 4,424 |
|  |  |  | |  |  | |  | |  |
| Test Less +Less•Late-2020=0 (p-value) | <0.001 | <0.001 | | <0.001 | <0.001 | | <0.001 | | <0.001 |
|  |  |  | |  |  | |  | |  |
| AIC | 26891.56 | 26688.24 | | 31566.45 | 31383.92 | | 26755.98 | | 26595.55 |
| BIC | 26932.69 | 26818.4 | | 31607.47 | 31513.75 | | 26797.03 | | 26725.48 |
| Notes: a) Regression coefficients with standard error in parenthesis below coefficient, *** p<0.001, ** p<0.01, * p<0.05. b) The original scale of life satisfaction is reversed in the analyses. | | | | | | | | | |
|  | | |  | | |  | |  | |

Table S3 Results from the linear mixed (multilevel) change score regressions for life satisfaction, loneliness, and psychological distress, adjusted and unadjusted for covariates for age group 50-64 years^a)^.

|  | Life satisfaction^b)^ | | Loneliness | | Psychological distress | |
| --- | --- | --- | --- | --- | --- | --- |
|  | Unadj. | Adjusted | Unadj | Adjusted | Unadj | Adjusted |
|  |  |  |  |  |  |  |
| Social contact=Less (ref.=More/Unchanged) | 0.12* | 0.092 | 0.21** | 0.23** | 0.13** | 0.10* |
|  | (0.049) | (0.049) | (0.072) | (0.073) | (0.040) | (0.041) |
| Time=Late-2020 (ref.=Mid-2020) | 0.39*** | 0.40*** | 0.52*** | 0.50*** | 0.35*** | 0.37*** |
|  | (0.048) | (0.048) | (0.070) | (0.070) | (0.039) | (0.039) |
| Interaction: Less social contact • Late-2020 | 0.27*** | 0.28*** | 0.24** | 0.24* | 0.17*** | 0.18*** |
|  | (0.064) | (0.064) | (0.094) | (0.094) | (0.052) | (0.052) |
| Sex=Male (ref.=Female) |  | -0.19*** |  | -0.050 |  | -0.11** |
|  |  | (0.044) |  | (0.067) |  | (0.040) |
| Partner=Non-cohab. partner (ref.=Married/Cohab.) |  | -0.31*** |  | -0.18 |  | -0.12 |
|  |  | (0.089) |  | (0.14) |  | (0.079) |
| Partner=Single (ref.= Married/Cohab.) |  | -0.19*** |  | 0.38*** |  | -0.11* |
|  |  | (0.056) |  | (0.086) |  | (0.051) |
| Education=Medium (ref.=Low) |  | -0.10 |  | -0.085 |  | 0.051 |
|  |  | (0.070) |  | (0.11) |  | (0.064) |
| Education=High (ref.=Low) |  | -0.083 |  | -0.11 |  | 0.073 |
|  |  | (0.070) |  | (0.11) |  | (0.063) |
| Employment=Temp.laid off (ref.=Employed) |  | 0.96*** |  | 0.25 |  | 0.70*** |
|  |  | (0.15) |  | (0.23) |  | (0.13) |
| Employment=Unemployed (ref.=Employed) |  | 0.12 |  | 0.37 |  | -0.14 |
|  |  | (0.19) |  | (0.29) |  | (0.16) |
| Employment=Pupil/Student (ref.=Employed) |  | -0.34 |  | 0.29 |  | 0.39 |
|  |  | (0.44) |  | (0.65) |  | (0.38) |
| Employment=Other (ref.=Employed) |  | -0.13** |  | -0.0073 |  | -0.12** |
|  |  | (0.048) |  | (0.074) |  | (0.043) |
| Home office=Yes (ref.=No) |  | -0.0093 |  | -0.13 |  | 0.076 |
|  |  | (0.052) |  | (0.077) |  | (0.044) |
| Leisure activities=1-3 times a month (ref.=Weekly) |  | 0.059 |  | -0.0061 |  | 0.072 |
|  |  | (0.068) |  | (0.10) |  | (0.062) |
| Leisure activities=Less often (ref.=Weekly) |  | -0.0024 |  | -0.19* |  | -0.0068 |
|  |  | (0.055) |  | (0.085) |  | (0.050) |
| Leisure activities=Never (ref.=Weekly) |  | -0.18** |  | -0.20* |  | -0.14* |
|  |  | (0.062) |  | (0.094) |  | (0.056) |
| Constant | -0.12** | 0.17 | -0.30*** | -0.12 | -0.41*** | -0.35*** |
|  | (0.040) | (0.086) | (0.060) | (0.13) | (0.034) | (0.077) |
|  |  |  |  |  |  |  |
| Observations | 8,439 | 8,385 | 8,140 | 8,088 | 8,323 | 8,267 |
| Number of groups | 5,060 | 5,036 | 4,977 | 4,954 | 4,996 | 4,974 |
|  |  |  |  |  |  |  |
| Test Less +Less•Late-2020=0 (p-value) | <0.001 | <0.001 | <0.001 | <0.001 | <0.001 | <0.001 |
|  |  |  |  |  |  |  |
| AIC | 32007.23 | 31715.21 | 37144.09 | 36880.39 | 28849.57 | 28616.27 |
| BIC | 32049.48 | 31848.86 | 37186.12 | 37013.36 | 28891.73 | 28749.65 |
| Notes: a) Regression coefficients with standard error in parenthesis below coefficient, *** p<0.001, ** p<0.01, * p<0.05. b) The original scale of life satisfaction is reversed in the analyses. | | | | | | |

Table S4 Results from the linear mixed (multilevel) change score regressions for life satisfaction, loneliness, and psychological distress, adjusted and unadjusted for covariates for age group 65+ years^a)^.

|  | Life satisfaction^b)^ | | Loneliness | | Psychological distress | |
| --- | --- | --- | --- | --- | --- | --- |
|  | Unadj. | Adjusted | Unadj | Adjusted | Unadj | Adjusted |
|  |  |  |  |  |  |  |
| Social contact=Less (ref.=More/Unchanged) | 0.073 | 0.041 | 0.18* | 0.18* | 0.039 | 0.031 |
|  | (0.058) | (0.059) | (0.090) | (0.091) | (0.045) | (0.046) |
| Time=Late-2020 (ref.=Mid-2020) | 0.37*** | 0.37*** | 0.56*** | 0.55*** | 0.31*** | 0.32*** |
|  | (0.054) | (0.054) | (0.084) | (0.084) | (0.041) | (0.042) |
| Interaction: Less social contact • Late-2020 | 0.36*** | 0.36*** | 0.23* | 0.25* | 0.20*** | 0.20*** |
|  | (0.076) | (0.076) | (0.12) | (0.12) | (0.058) | (0.059) |
| Sex=Male (ref.=Female) |  | -0.33*** |  | -0.13 |  | -0.18*** |
|  |  | (0.055) |  | (0.082) |  | (0.044) |
| Partner=Non-cohab. partner (ref.=Married/Cohab.) |  | -0.035 |  | 0.083 |  | -0.077 |
|  |  | (0.12) |  | (0.18) |  | (0.093) |
| Partner=Single (ref.= Married/Cohab.) |  | -0.10 |  | 0.45*** |  | 0.091 |
|  |  | (0.067) |  | (0.10) |  | (0.054) |
| Education=Medium (ref.=Low) |  | -0.041 |  | -0.021 |  | 0.037 |
|  |  | (0.076) |  | (0.12) |  | (0.060) |
| Education=High (ref.=Low) |  | 0.0029 |  | -0.048 |  | 0.070 |
|  |  | (0.072) |  | (0.11) |  | (0.058) |
| Employment=Temp.laid off (ref.=Employed) |  | 0.83 |  | -0.47 |  | 0.86* |
|  |  | (0.49) |  | (0.73) |  | (0.38) |
| Employment=Unemployed (ref.=Employed) |  | -0.089 |  | 0.91 |  | 1.77*** |
|  |  | (0.65) |  | (0.97) |  | (0.50) |
| Employment=Pupil/Student (ref.=Employed) |  | -1.87 |  | - |  | 1.30 |
|  |  | (1.40) |  | - |  | (1.08) |
| Employment=Other (ref.=Employed) |  | -0.032 |  | 0.082 |  | 0.14 |
|  |  | (0.089) |  | (0.13) |  | (0.071) |
| Home office=Yes (ref.=No) |  | -0.051 |  | 0.051 |  | 0.42*** |
|  |  | (0.15) |  | (0.22) |  | (0.12) |
| Leisure activities=1-3 times a month (ref.=Weekly) |  | -0.081 |  | 0.15 |  | 0.053 |
|  |  | (0.078) |  | (0.12) |  | (0.063) |
| Leisure activities=Less often (ref.=Weekly) |  | -0.084 |  | -0.10 |  | 0.000091 |
|  |  | (0.068) |  | (0.10) |  | (0.054) |
| Leisure activities=Never (ref.=Weekly) |  | -0.22** |  | -0.053 |  | -0.0024 |
|  |  | (0.076) |  | (0.11) |  | (0.061) |
| Constant | 0.056 | 0.41** | -0.048 | -0.095 | -0.19*** | -0.29** |
|  | (0.048) | (0.13) | (0.073) | (0.19) | (0.037) | (0.100) |
|  |  |  |  |  |  |  |
| Observations | 5,211 | 5,134 | 4,798 | 4,737 | 5,168 | 5,094 |
| Number of groups | 3,083 | 3,049 | 2,942 | 2,908 | 3,061 | 3,030 |
|  |  |  |  |  |  |  |
| Test Less +Less•Late-2020=0 (p-value) | <0.001 | <0.001 | <0.001 | <0.001 | <0.001 | <0.001 |
|  |  |  |  |  |  |  |
| AIC | 19249.63 | 18931.91 | 21329.15 | 21033.82 | 16527.61 | 16251.23 |
| BIC | 19288.99 | 19056.24 | 21368.01 | 21150.16 | 16566.92 | 16375.41 |
| Notes: a) Regression coefficients with standard error in parenthesis below coefficient, *** p<0.001, ** p<0.01, * p<0.05. b) The original scale of life satisfaction is reversed in the analyses. | | | | | | |
